# Work-relatedness of inguinal hernia: a systematic review including meta-analysis and GRADE

**DOI:** 10.1007/s10029-020-02236-0

**Published:** 2020-05-30

**Authors:** P. P. F. M. Kuijer, D. Hondebrink, C. T. J. Hulshof, H. F. Van der Molen

**Affiliations:** Netherlands Center for Occupational Diseases, Coronel Institute of Occupational Health, Amsterdam Public Health Research Institute, Amsterdam Movement Sciences, Amsterdam UMC, Location AMC, University of Amsterdam, Amsterdam, The Netherlands

**Keywords:** Occupational disease, Risk factors, Occupational exposure, Etiology, Prevention

## Abstract

**Purpose:**

Clinicians need to know whether inguinal hernia (IH) can be attributed to work to answer questions regarding prevention and medical causation. This review describes whether work-related risk factors are associated with IH.

**Methods:**

A systematic review was performed in Medline via PubMed until February 3rd, 2020. Inclusion criteria were that IH was diagnosed by a clinician, and workers exposed to work-related risk factors were compared to workers less exposed or not at all. A quality assessment and a meta-analysis using Cochrane’s RevMan 5.3 were performed, including GRADE for quality of evidence.

**Results:**

The search resulted in 540 references. Fourteen studies fulfilled the inclusion criteria, of which three were included in a meta-analysis, all three being of high quality, including 621 workers diagnosed with IH. The meta-analysis revealed significant associations with physically demanding work (OR 2.30, 95% CI 1.56–3.40). Two prospective studies, including 382 and 22,926 cases revealed associations that this was true for male workers with a lateral IH that reported standing or walking for more than six hours per workday (OR 1.45, 95% CI 1.12–1.88) or lifting cumulative loads of more than 4000 kg per workday (OR 1.32, 95% CI 1.27–1.38). The level of certainty for the latter two work-related risk factors was moderate and high according to GRADE.

**Conclusion:**

Lateral IH among males is associated with work-related risk factors depending on the level of exposure to the time standing/walking per workday, or the amount of load lifted per workday.

**Electronic supplementary material:**

The online version of this article (10.1007/s10029-020-02236-0) contains supplementary material, which is available to authorized users.

## Introduction

Worldwide, more than 20 million patients undergo groin hernia repair annually, a vast majority being male workers of working age [1]. The international guidelines for groin hernia management established that risk factors for primary inguinal hernia (IH) among adults include: family history, previous contra-lateral hernia, male sex, age, abnormal collagen metabolism, prostatectomy, and low body mass index [1]. Regarding work, the conclusion was that contradictory evidence existed that social class, occupational factors, and workload affect the risk of IH repair [1–3], and that heavy lifting may predispose to IH formation [1, 4]. The search for the international guidelines for groin hernia management was conducted until July 2015 [1]. Since then, new prospective cohort studies have been published that might alter the inconclusive evidence regarding the effect of work [5, 6]. Moreover, no meta-analysis has yet been performed to substantiate the evidence.

This knowledge regarding the work-relatedness of IH is of importance for patients and clinicians in order to answer questions regarding prevention and medical causation [7, 8]. For prevention, a prerequisite is knowing whether work-related risk factors actually do matter in the onset or worsening of a disease [9]. In addition, if data allow, clinically relevant exposure threshold limits can be formulated, as is done for several other prevalent diseases like carpal tunnel syndrome [10], lateral epicondylitis [11], specific shoulder disorders [12], hip and knee osteoarthritis [13, 14], and lumbosacral radiculopathy syndrome [15]. Regarding medical causation, many countries provide financial compensation when a disease is recognised as an occupational disease, like the Unites States of America, Canada and many countries in the European Union, like Italy, France, and Germany. Therefore, the aim of this systematic review is to assess to what extent work-related risk factors are associated with clinically assessed IH among workers.

## Methods

The systematic review and meta-analysis were performed in line with the criteria of the PRISMA statement [16] (Online Appendix I). No review protocol was published beforehand.

### Eligibility criteria

The following inclusion criteria were used: the study was written in English or German; the study presented original data; participants were workers; IH was diagnosed by a clinician without taking into account the diagnostics tests used; work-related risk factors were described in terms of, for instance, type of industry, job or occupation, physical workload, or specific occupational activities like lifting. To obtain a good overview of the data, all study designs and all follow-up periods were included as long as the data were described in terms of IH being present or not, and exposure was described in terms of exposed versus less exposed or non-exposed.

### Search and source

A systematic literature search was performed in Medline using Pubmed, until February 3rd, 2020. The search strategy involved combining searches for the disease IH, terms for work-related exposure, and for epidemiological studies on risk factors. In addition, the references of the included studies were screened and personal files of the authors for additional studies. Table 1 shows the search strategy.

**Table 1:** Search strategy in Medline using PubMed performed February 3rd, 2020

	PubMed
Worker	((adult[mesh]) OR (adult[tiab]) OR (middle aged[mesh]) OR (middle aged[tiab]))
Inguinal hernia	((inguinal hernia[mesh]) OR (inguinal hernia[tiab]))
Work-related etiology	((occupational disease*[mesh]) OR (occupational disease*[tiab]) OR (risk factor*[mesh]) OR (risk factor*[tiab]) OR (work-related[tiab]) OR (etiology[mesh]) OR (etiology[tiab]))

### Study selection

After duplicates from PubMed were removed, two reviewers (PK, DH) independently checked the fulfilment of the inclusion criteria. We first screened titles and abstracts and excluded studies that did not fulfil the inclusion criteria. Of the remaining references, we obtained the full text and assessed them independently for eligibility based on the full texts. Any disagreements by the two reviewers were resolved through discussion and if necessary, a third assessor (HvdM) made the final decision.

### Data collection

The following data were extracted by one author (DH) and independently checked by a second author (PK): author, year of publication, study design; case definition of IH; source of retrieving participants; number and characteristics of participants like sex and age; exposure definition; number of workers with or without IH for the described exposure categories; (adjusted) risk estimate like Odds Ratios (OR) and 95% confidence intervals (95% CI) (Online Appendix II).

### Quality assessment

For the quality assessment of each study, a total of 16 items across five categories were assessed by two reviewers independently (PK, DH) [12]. The five categories were: (1) study population (three items, for instance positive if the participation of both the exposed and unexposed groups was ≥ 70%); (2) assessment exposure (three items, for instance whether the exposure was assessed by an independent person and not based on self-report); (3) assessment outcome (three items, for instance whether IH was diagnosed by a clinician); (4) study design (four items, for instance whether the follow-up period was ≥ 1 year); and (5) data analysis (three items, for instance whether the method used to control for confounding was described). Each item was scored as ‘positive’, ‘negative’, or ‘unclear’. Differences in outcome were mutually discussed until consensus was reached. High quality was defined as 11 or more items being rated as ‘positive’ out of a total of 16 criteria [12]. The quality assessment was performed for studies used in the meta-analyses regarding physical workload and/or specific occupational activities.

### Data analysis

To answer the research question, a meta-analysis was performed to establish whether risk factors were sufficiently homogeneous across at least two studies. For each risk factor, the highest versus the lowest exposures as reported in the studies were used. We calculated a pooled OR and 95% CI for each risk factor, including *I*^2^ as measure of consistency, using a random effects model in Cochrane’s RevMan 5.3, for both the high and low risk of bias studies combined and only for the studies with a low risk of bias, if possible. The results are presented as forest plots including the contribution of each study (weight) to the overall effect (Mantel–Haenszel, random) using RevMan 5.3. No additional statistical analyses were performed.

## GRADE

GRADE (Grades of Recommendations, Assessment, Development and Evaluation) was used to assess the quality of evidence for the studies used in the meta-analyses regarding physical workload and/or specific occupational activities. The criteria of the framework for prognostic studies were used [17]. Four levels of quality were used: high, moderate, low, and very low. Therefore, our starting point for the quality of the evidence was ‘moderate’ for prospective explanatory cohort studies, given the inclusion of only studies specifically focusing on work-related risk factors for IH. Next, the quality of evidence was downgraded based on the following five factors: (1) study strengths (majority of studies having high risk of bias or minority of studies having a prospective study design); (2) consistency (*I*^2^ > 70%); (3) indirectness (a priori not true, given our inclusion criteria that IH is diagnosed by a physician and the population of interest is workers); (4) imprecision (less than 95 IH patients included or 95% confidence interval of the effect size of the studied risk factor includes 1, unless the boundaries of the lower and upper limit of the 95% confidence interval are smaller than 0.8–1.2, indicating high certainty of no effect of the studied risk factor for IH); and (5) publication bias (Yes). Finally, study findings with effect sizes (i.e. lower limit of 95%CI Risk estimate > 2.0) and the presence of an exposure–response relationship in the majority of studies (Yes) resulted in an upgrade of the quality of evidence.

## Results

### Types of studies and quality

**Fig. 1:**
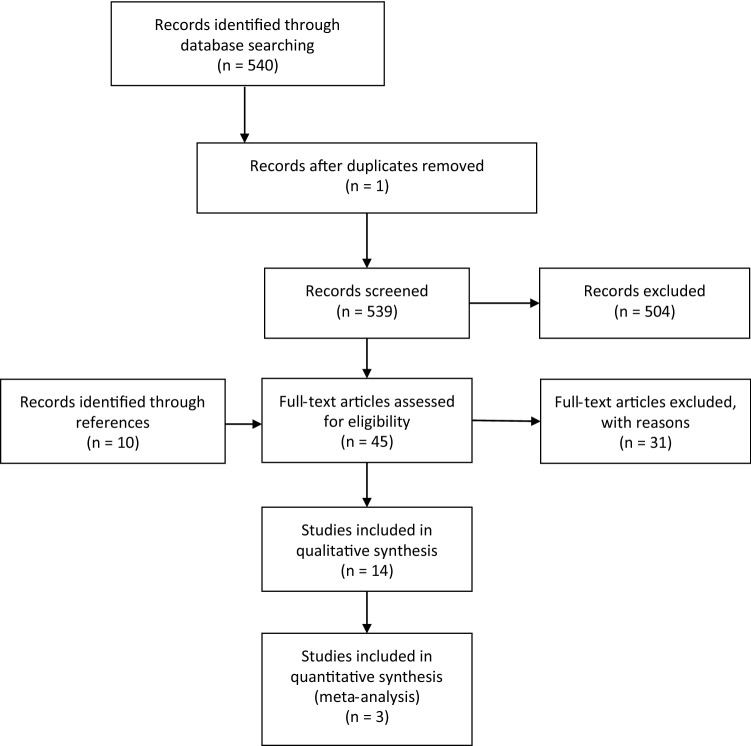
Flowchart of the included studies

The search resulted in a total of 540 references, of which 14 fulfilled the inclusion criteria [3, 4, 6, 18–28] (Fig. 1, Online Appendix II). Two studies were case reports [26, 27], one had a cross-sectional design [18], five a case control design [4, 19, 20, 22, 23], one a retrospective cohort design [28] and five a prospective cohort design [3, 6, 21, 24, 25]. Five studies assessed the association between IH and a short-term occupational exposure (single strenuous work-related event) [24–28]. Nine studies assessed the long-term occupational exposure: one study assessed the type of industry and jobs involved [18], six the physical workload [4, 19–23], and two specific occupational activities, namely standing/walking and lifting [3, 6] (Online Appendix II). Three of the studies on physical workload were able to be used for the meta-analyses, given sufficient data reported about the presence of IH among exposed versus less exposed workers performing physically demanding work [4, 21, 23]. The three studies in the meta-analyses and two studies on specific occupational activities were all of high quality with scores varying between 12 and 15, out of a maximum score of 16 (Table 2). The three criteria with the lowest score were: (1) participation rates < 70%; (2) no prospective study design; and (3) insufficient data about completers versus withdrawals.

**Table 2:**
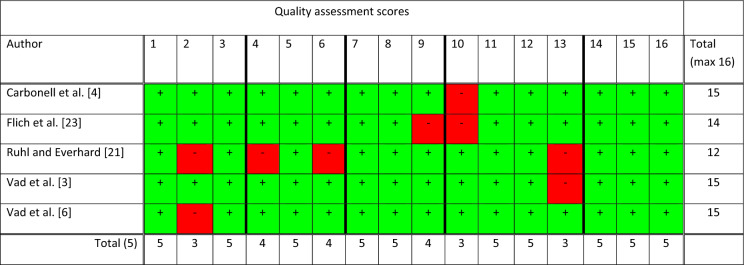
Quality assessment of the studies on work-related risk factors graded as positive ( +) or negative (−) according to 16 items

Study population: 1. Study groups defined, 2. Participation ≥ 70%, 3. Number case ≥ 50; Assessment of exposure: 4. Exposure measurement, 5. Dose–response, 6. Blind for outcome status; Assessment outcome: 7. Outcome definition, 8. Assessment method, 9. Blind for exposure status; Study design: 10. Longitudinal, 11. Inclusion and exclusion criteria, 12. Follow-up period ≥ 1 year, 13. Info completers versus withdrawals; Data analysis: 14. Data presentation, 15. Consideration of confounders, 16. Control for confounding

### Physically demanding work

**Fig. 2:**
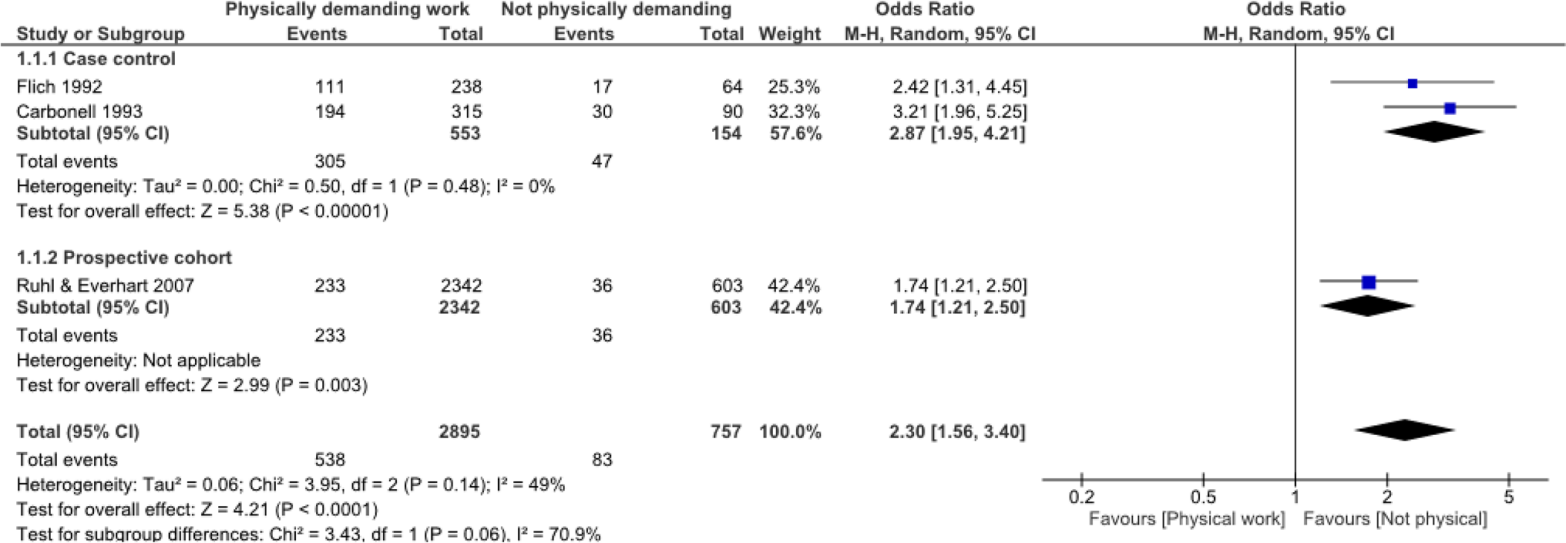
Forest plot of the association between physically demanding work and IH

The meta-analyses based on two high-quality case control studies [4, 23] and one high-quality prospective cohort study [21] showed that physically demanding work was associated with an increased risk for IH (OR 2.30, 95% confidence interval 1.56–3.40) (Fig. 2). Physically demanding work was based on: (1) the self-reported degree of effort of the work categorised into four categories (no, light, medium, and high, and comparing no and light versus medium and high) [23]; (2) a summarised effort score varying between 1 and 10 based on ten interview questions regarding for instance the number of days physically demanding work is performed; the number of times heavy objects are lifted, and whether the work is performed sitting or standing (comparing a score 0–2.5 versus 5.0–10.0) [4]; (3) and a self-report regarding non-recreational physical activity into three categories (low, moderate, and high) and comparing low versus high [21] (Online Appendix II).

### Occupational activities

Vad et al. [3, 6] studied the exposure to the occupational activities of standing/walking and lifting for IH in two prospective cohort studies including 40,395 and 696 IH patients, respectively (Online Appendix II). The exposure to standing/walking and lifting was assessed using information from a Job Exposure Matrix based on expert judgements and year-by-year information on Danish International Standard Classification of Occupations codes for each individual. Due to differences in the definition of exposure categories and potential overlap in similar cases, the data could not be pooled. Their first finding was that these occupational activities were only associated with lateral IH in male workers and not medial IH. Next, standing or walking for more than six hours per workday was associated with IH (HR = 1.45; 95% CI 1.12–1.88, adjusted for age, body mass index (BMI), leisure time physical activity and smoking, and other factors) and for lifting more than 4000 kg per workday (OR 1.32; 95% CI 1.27–1.38, adjusted for age, socioeconomic position and country region).

## GRADE

The evidence for the work-related risk factor of physically demanding work was rated as low (Table 3). The level of evidence was downgraded because two of the three studies had no prospective design, shifting the overall quality from ‘moderate’ to ‘low’. In addition, no upgrades were present. In GRADE terminology, this means: ‘Our confidence in the effect estimate is limited. The true effect may be substantially different from the estimated increased risk of 2.30 for IH due to performing physically demanding work’. In contrast, neither of the studies of Vad et al. [3, 6] had a downgrade. Vad et al. [3] also had one upgrade for the presence of a dose–response relationship [3], thereby shifting the evidence from ‘moderate’ to ‘high’. In GRADE terminology, this means that ‘we are moderately confident about the increased risk of 1.45 for a lateral IH due to standing or walking for more than six hours per workday: the true effect is likely to be close to the estimate of the effect, but there is a possibility that it is different’. For lifting, this means that ‘we are very confident that the true effect lies close to that of the estimated increased risk of 1.32 for a lateral IH due to lifting more than 4000 kg per workday’.

**Table 3:** GRADE framework for the work-related risk factors in terms of physically demanding work and specific occupational activities for IH

Work-related risk factor	Risk of bias		Inconsistency Heterogeneity (*I*^2^ > 70%)	Indirectness		Imprecision		Publication bias Strongly suspected	Down grade?	Lower limit 95%CI risk > 2.0	Dose- response present	Certainty
	#Studies with high risk of bias	#Prospective cohort studies		Diagnosis physician	Workers	# Cases (< *n* = 95)	Significant effect (risk, 95% CI)					
Physically demanding work [4, 21, 23]	0/3	1/3**↓**	49%	Yes	Yes	621 IH	2.30 (1.56–3.40)	Not applicable	Yes	Not applicable	Not applicable	Low
Stand/walk ≥ 6 h/day [6]	0/1	1/1	Not applicable	Yes	Yes	382 lateral IH	1.45 (1.12–1.88)	Not applicable	No	1.12	No	Moderate
Lift > 4000 kg/day [3]	0/1	1/1	Not applicable	Yes	Yes	22,926 lateral IH	1.32 (1.27–1.38)	Not applicable	No	1.27	Yes, *p* < 10^–3^**↑**	High

## Discussion

### Work-related disease

This review shows that a lateral IH in male workers is associated with physically demanding work that is characterised by standing/walking and lifting. The evidence for both risk factors was of moderate and high quality in GRADE terms, respectively. This review substantiates four of the Hill criteria for causality [29], namely: (1) temporality—the majority of studies had a prospective design; (2) consistency—all five high-quality studies showed that a physical workload was a risk factor [3, 4, 6, 21, 23]; (3) strength—the presence of a dose–response relationship for lifting [3]; and (4) specificity—this appeared true for only a lateral IH [3, 6]. To substantiate a fifth criterion for causality, namely plausibility, studies are needed that show that an increased abdominal pressure while standing/walking or lifting results in a protrusion of the abdominal content through the inguinal canal, while sitting may prevent this [3, 6]. Unfortunately, no papers have been found that studied the effect of prevention at work sites to reduce the incidence of lateral IH among male workers, preferably by reducing the time spent walking/standing or by reducing the workload due to lifting. If such studies had been performed and indeed had established a preventive effect, this might have substantiated a sixth factor for causality, namely coherence. Based on recently performed cohort studies, a clinically relevant threshold limit for standing/walking might be a maximum of 4 h per workday, and for lifting a maximum of 1000 kg per workday [3, 6]. So, based on this review and the above line of reasoning, a future update of the international guidelines for groin hernia management might take into account work as a risk factor for the onset or worsening of lateral IH in males [1].

### Strengths and limitations

A strength of the present review is that the results are based on five high-quality studies including a meta-analysis and the application of GRADE. A second strength of our study is that only clinically assessed IH patients were included. A third strength is that both studies by Vad et al. [3, 6] took into account age as confounder. Moreover, Vad et al. [6] took also into account the personal risk factors BMI, leisure-time physical activity and smoking status, although leisure-time physical activity and smoking status showed no association with lateral (or medial) IH repair in their cohort. This is in line with the reported personal risk factors in international guidelines for groin hernia management [1].

A limitation of our study is that exposures are based on self-reports [4, 21, 23] and expert judgements [3, 6]. Unfortunately, expert judgements and self-reports provide only limited insight into the occurrence of activities [30]. Future studies, preferably on the effectiveness of prevention for lateral IH among male workers, should gather information from measurements on the time spent walking/standing per workday or data about productivity regarding the amounts of load lifted per workday. Finally, as described in Online Appendix II, the studies by Vad [3, 6] used both data from a similar Danish cohort of first-time inguinal hernia repairs in the period of 1998–2008. To prevent potential overlap due to similar patient data, the two studies were not pooled and for the GRADE assessment only the study with the clearest exposure definition for practice was used.

## Electronic supplementary material

Below is the link to the electronic supplementary material.

Supplementary file1 (DOC 64 kb)

Supplementary file2 (DOCX 55 kb)

## Data Availability

All data are available and retrievable via PubMed.
