# Supplementary material for: Work-relatedness of inguinal hernia: a systematic review including meta-analysis and GRADE
Source: Hernia. 2020 May 30;24(5):943–50. doi: 10.1007/s10029-020-02236-0 (PMC7520410; doi:10.1007/s10029-020-02236-0)
Supplement: Supplementary file 2 — Supplementary file2 (DOCX 55 kb) [file 10029_2020_2236_MOESM2_ESM.docx]

Appendix II Data extraction table of the included studies categorised according to type of industry or job, physical workload and specific occupational activities

| **Author and study design** | **Population** | **Risk factor** | **Definition outcome, exposed versus less or non-exposed** | **Risk estimate** |
| --- | --- | --- | --- | --- |
| **Type of industry or job** | | | | |
| Kang [18]  Cross-sectional | The injury and illness data are obtained from Occupational  Safety and Health Administration's (OSHA) 200 logs  and supplementary records that establishments maintain  throughout the reference year [U.S. Department of Labor,  1994]. The survey excludes self-employed people, farms  with fewer than 11 employees, private households, and  Federal, State, and local government agencies. Data for  mines and railroads are provided to the BLS by the Department of Labor's Mine Safety and Health Administration  and the Department of Transportation's Federal Railroad Administration, and are reported with the Annual  Survey results.  N = 51246000 male workers.  There were 30791 cases. | Working in a certain industry or having a certain type of job. | Incidence rate:  Incidence per 10000 workers.  Rate ratio:  Hernia incidence rate (IR) for each occupation divided by the hernia incidence rate of total workers (reference). | *Hernia by industry*  Total (reference)(N males, N cases):   - 51246000, 30791   IR = 6.0  Rate ratio (95% CI) = 1.00 (0.82-1.18)  Agriculture:   - 1370000, 516   IR = 3.8  Rate ratio = 0.63  Mining:   - 549000, 271   IR = 4.9  Rate ratio = 0.82  Construction:   - 4908000, 4129   IR = 8.4  Rate ratio = 1.40  Manufacturing:   - 13337000, 11810   IR = 8.9  Rate ratio = 1.47  Transportation:   - 4745000, 2391   IR = 5.0  Rate ratio = 0.84  Wholesale trade:   - 3074000, 3294   IR = 10.7  Rate ratio = 1.78  Retail trade:   - 9379000, 4681   IR = 5.0  Rate ratio = 0.83  Finance and insurance   - 2819000, 620   IR = 2.2  Rate ratio = 0.37  Service   - 11047000, 3080   IR = 2.8  Rate ratio = 0.46  *Hernia by occupation*  Handlers, equipment cleaners, helpers and labourers (N males, N cases):   - 3785000, 5661   IR = 15.0  Rate ratio (95% CI) = 2.47 (2.14-2.80)  Machine operators and tenders:   - 2966000, 3824   IR = 12.9  Rate ratio (95% CI) = 2.13 (1.81-2.44)  Fabricators, assemblers and handworking occupations:   - 1290000, 1585, 12.3   IR = 12.3  Rate ratio (95% CI) = 2.03 (1.69-2.37)  Mechanics and repairers:   - 3486000, 3623   IR = 10.4  Rate ratio (95% CI) = 1.72 (1.43-2.00)  Transportation and material moving equipment occupations:   - 3900000, 3410, 8.7, 1.44 (1.18-1.71)   IR = 8.7  Rate ratio (95% CI) = 1.44 (1.18-1.71)  Production inspectors, testers, samplers and weighers:   - 340000, 271, 8.0, 1.32 (0.95-1.69)   IR = 8.0  Rate ratio (95% CI) = 1.32 (0.95-1.69)  Extractive occupations:   - 138000, 106   IR = 7.7  Rate ratio (95% CI) = 1.21 (0.83-1.71)  Construction trade:   - 3557000, 2735   IR = 7.7  Rate ratio (95% CI) = 1.27 (1.02-1.52)  Precision product occupations:   - 2692000, 1716, 6.4   IR = 6.4  Rate ratio (95% CI) = 1.05 (0.81-1.30)  Administrative support:   - 2816000, 1533, 5.4   IR = 5.4  Rate ratio (95% CI) = 0.90 (0.67-1.12)  Service except private household and protective services:   - 4141000, 2201, 5.3   IR = 5.3  Rate ratio (95% CI) = 0.88 (0.66-1.09)  Agricultural occupations:   - 1464000, 689, 4.7   IR = 4.7  Rate ratio (95% CI) = 0.78 (0.54-1.02)  Technicians and related support occupations:   - 1478000, 516   IR = 3.5  Rate ratio (95% CI) = 0.58 (0.36-0.79)  Protective services:   - 575000, 160   IR = 2.8  Rate ratio (95% CI) = 0.46 (0.22-0.70)  Sales occupations:   - 6385000, 1584   IR = 2.5  Rate ratio (95% CI) = 0.41 (0.26-0.56)  Professional specialty occupations:   - 5225000, 617, 1.2, 0.20 (0.07-0.32)   IR = 1.2  Rate ratio (95% CI) = 0.20 (0.07-0.32)  Executive, administrative and specialty occupations:   - 6984000, 570   IR = 0.8  Rate ratio (95% CI) = 0.13 (0.03-0.24)  *Top 10 high-risk occupations among 40 major occupations*  Labourers, non-construction (n males, n cases):   - 919000, 2495   IR = 27.2  Rate ratio (95% CI) = 4.52 (4.04-5.00)  Miscellaneous machine operators:   - 745000, 1271   IR = 17.1  Rate ratio (95% CI) = 2.84 (2.42-3.26)  Plumbers and pipefitters:   - 388000, 630   IR = 16.2  Rate ratio (95% CI) = 2.70 (2.19-3.21)  Construction labourers:   - 650000, 915   IR = 14.1  Rate ratio (95% CI) = 2.34 (1.94-2.74)  Freight, stock, material handlers:   - 607000, 818   IR = 13.5  Rate ratio (95% CI) = 2.24 (1.85-2.64)  Welders and cutters:   - 517000, 674   IR = 13.0  Rate ratio (95% CI) = 2.17 (1.77-2.57)  Assemblers:   - 685000, 843   IR = 12.3  Rate ratio (95% CI) = 2.05 (1.67-2.43)  Shipping and receiving clerks:   - 404000, 495   IR = 12.3  Rate ratio (95% CI) = 2.04 (1.63-2.45)  Truck drivers:   - 2328000, 2451   IR = 10.5  Rate ratio (95% CI) = 1.75 (1.45-2.05)  Janitors and cleaners:   - 921000, 901   IR = 9.8  Rate ratio (95% CI) = 1.63 (1.29-1.96) |
| Physical workload | | | | |
| Ashindoitiang, et al. [22]  Case control | All male patients aged 18 years and above who presented with primary inguinal  hernia at the general surgical clinic of Ikorodu General Hospital between April 2009 and March 2011 were accepted into the study as cases.  N = 404 male patients     - Case group:   N = 202, age 16-80.     - Control group: N = 202, age 17-83. | Strenuous activities are classified as vigorous (metabolic  equivalent of task (MET) > 6.0).  Non-strenuous activities are classified as moderate (metabolic equivalent of task (MET) between 3.0 and 6.0). | Strenuous activities present vs. absent (reference).  Strenuous activities present vs. absent (reference), adjusted for: chronic cough, straining during urination/defecation, ascites/abdominal mass, family history of inguinal hernia, obesity and smoking. | Strenuous activities absent (reference) (N = cases, controls):   - 86 - 160   Strenuous activities present (N = cases, controls):   - 116 - 42   Univariate OR (95% CI):   - 5.138 (3.331-7.975)   Multivariate OR (95% CI):   - 5.988 (3.667-9.777)   *Type of hernia*  Strenuous activities present (N = indirect (%), direct (%)):   - 69 (52%) - 47 (67%)   Strenuous activities absent (N = indirect (%), direct (%)):   - 63 (48%) - 23 (33%) |
| Carbonell et al. [4]  Case control | The cases were 290 patients operated on for inguinal (direct and indirect) and femoral hernias from 1987-1989 at the hospital Peset Aleixandre.  Controls were selected randomly from the same population.  N = 580 people.    Case group:  N = 290, age 21-86.   - 228 ♂ - 62 ♀     Control group:  N = 290, age 21-90.   - 228 ♂ - 62 ♀ | An effort score of 1-10, for a maximum of four different activities or jobs before the hernia developed, was calculated using the following variables:   - Sedentary work (score 1) or standing still (score 2). - Number of hours/day spent standing. - Number of times heavy objects were lifted. - Weight of these objects (kg). - Number of days/weeks of physical exertion. - Number of days/weeks doing labourers’ work. - Number of years spent doing each activity. - Physical exertion only at work (if yes score 1, if not score 0). - Physical exertion only at play (same as above). - Physical exertion at work and at play (same as above).   The effort score ranged from 1 to 10, after logarithmically converting the multiplied value of each variable. | An effort score of 1 (reference) was compared to higher effort scores across different jobs. | *Risk factors for inguinal hernia*  Effort needed for first job (RR, 95% CI):   - RR = 2.92 (3.667-9.777)   P < 0.01  Effort needed for second job (RR, 95% CI):   - RR = 2.47 (1.70-3.58)   P = 0.09  Effort needed for third job (RR, 95% CI):   - RR = 3.22 (1.64-6.31)   P = 0.71  Effort needed for fourth job (RR, 95% CI):   - RR = 1.71 (0.41-7.03)   P = 0.99 |
| Dickerman et al. [26]  Case report | N = 1, a 42-year-old male powerlifter who was seen for complaints of excessive oesophageal reflux, an umbilical hernia and bilateral inguinal hernias. | Wearing a powerlifting belt, since the inguinal hernias occurred after the umbilical hernia while the patient was wearing a belt. | N/A | According to the study, there is no doubt the subject’s competitive powerlifting career predisposed him to hernia formation. Even though no reports exist on an increased incidence of inguinal or umbilical hernias developing in power athletes, they previously established the increased incidence of hiatal hernias in young powerlifters and thus propose that this case of multiple hernias is likely the result of a pressure-overload syndrome that can occur in competitive powerlifters. |
| Flich et al. [23]  Case control | The 128 cases were all patients with  inguinal hernias treated in 1986 in hospital (the General Hospital of Valencia). All were in-patients for surgery.  The 174 controls were selected from the same geographic area. They were randomly selected from the total population tabulated at the health care centre.  N = 302 cases and controls    N = 128 cases (mean age 50.23):   - 107 ♂ - 21 ♀     N = 174 controls (mean age 50.82):   - 151♂ - 23♀ | Physical activity, including intensity and length of time in activity, was split into four categories.  The inclusion of an effort category (each person can present different categories in the course of  his/her life), depended on the type of work carried out for the longest period of time.  The categories are as follows:  1) No effort, activities involving no effort and sedentary work were included;  2) light, standing work involving occasional lifting of not too heavy weights;  3) medium, when weight was lifted more frequently;  4) high, when the effort was daily. | Cases and controls (reference) in different effort categories (high + medium and no + light) were compared to each other.  Cases and controls (reference) in different effort categories (high and no) were compared to each other.  Cases and controls of different years of effort (1-10, 20-39 and 40-69) categories were compared to cases and control of the years of effort = 0 category (reference)  Cases and controls in the effort category of light + medium and high were categorised in different groups of years of effort (1-19, 29-39 and 40-69). These were compared to the years of effort = 0 (reference) | *Effort intensity*  Effort high + medium (N = cases, controls):   - 111 - 127   Effort no + light (N = cases, controls):   - 17 - 47   OR (95% CI*) = 2.41 (1.31-4.45)  x^2^/P = < 0.05  Effort high (N = cases, controls):   - 37 - 16   Effort no (N = cases, controls):   - 1 - 10   OR (95% CI*) = 23.12 (2.73-196.09)  x^2^/P = < 0.0001  *Time of exposure to effort*  Years of effort: 0 (N = cases, controls):   - 1 - 10   OR = 1.00  Years of effort: 1-10:   - 12 - 49   OR (95% CI*) = 2.45 (0.29-21.03)  Years of effort: 20-39:   - 40 - 50   OR (95% CI*) = 8.00 (0.98-65.15)  Years of effort: 40-69:   - 75 - 65   OR (95% CI*) = 11.54 (1.44-92.57)  P ≤ 0.01  *Categories and time of exposure to effort*  Effort light + medium  Years of effort: 1-19 (N = cases, controls):   - 22 - 55   OR (95% CI*)= 4.00 (0.48-33.13)  Years of effort: 29-39:   - 26 - 33   OR (95% CI*)= 7.87 (0.95-65.57)  Years of effort: 40-69:   - 41 - 36   OR (95% CI*)= 11.38 (1.39-93.36)  x^2^/P =<0.001  Effort high  Years of effort: 1-19 (N = cases, controls):   - 19 - 14   OR (95% CI*)= 13.57 (1.55-118.68)  Years of effort: 29-39:   - 13 - 2   OR (95% CI*)= 65.00 (5.14-822.63)  Years of effort: 40-69:   - 5 - 0   OR = Not available  * = self-calculated |
| Lau et al. [19]  Case control | Between January 2002 and January 2004, male patients who presented with primary inguinal hernia at the general surgical or hernia specialist clinic, University of Hong Kong Medical Center, were recruited as cases.  N = 1418 male subjects     - Case group: N = 709 cases with primary inguinal hernia.   Age 65 ± 13     - Control group: N = 709 age matched controls. | Physical activity was quantified in terms of three dimensions:  (1) physical activity at work,  (2) sports activity  during leisure time,  (3) physical activity during  leisure time excluding sport.  Subjects responded to a 5-point scale with descriptions ranging from never (point value 1) to always (point value 5). For each category, high activity was represented by a maximum score of 5 and low activity by a minimum  score of 1. The indices of physical activity, including scores for the work index, sport index, and leisure index, were calculated as the sum of intensity multiplied by time engagement. A total activity index was then derived by summing these 3 values. | Work and total activity indexes of cases and controls (reference) were compared. | Work activity index (range):   - Cases = 2.8 (2.3-3.3) - Controls = 2.7 (2.2-3.2)   P = 0.03  Total activity index (range):   - Cases = 7.7 (7.1-8.3) - Controls = 7.4 (6.8-8.0)   P = 0.01 |
| Liem et al. [20]  Case control | All incident female cases of inguinal hernia in six participating hospitals in the Netherlands, between January 1994 and November 1995, were collected and registered.  Controls were selected from females who visited the outpatient surgical clinic for excision of common benign tumours of the skin unlikely to confound a comparative analysis.  N = 197 female subjects, age 20-80     - Case group: N = 72      - Control group: N = 125 | For each category (work, sports activity and leisure time), high activity was represented by the maximum score of 5, and low activity by the minimum score of 1. A total activity index was calculated by adding the three separate scores.  Work activity in the past was estimated using four categories (sedentary, standing, labour, heavy labour) using examples for both activity level and job type. | The present work activity index, duration of present work, and the present leisure-time activity index of cases and controls (reference) were compared.  Also, duration of present work x present work activity index was compared between cases and controls (reference).  The past work activity index, duration of past work, and the past leisure-time activity index of cases and controls (reference) were compared.  Also, duration of past work x past work activity index was compared between cases and controls (reference). | Present work activity index (range)   - Cases = 2.9 (2.6-3.2) - Controls = 2.9 (2.6-3.1)   P = 0.6  Duration present work in years:   - Cases = 26 (17-43) - Controls = 30 (12-43)   P = 1.0  Duration x present work activity:   - Cases = 86.9 (43.6-118.1) - Controls = 86.6 (36.3-123.5)   P = 1.0  Present leisure-time activity index:   - Cases = 2.9 (2.7-3.3) - Controls = 3.0 (2.6-3.3)   P = 1.0  Present total index:   - Cases = 8.0 (7.2-8.5) - Controls = 8.1 (7.3-9.0)   P = 0.15  Past work activity index (range):   - Cases = 1 (0-3) - Controls = 2 (0-3)   P = 0.9  Duration past work in years:   - Cases = 5 (0-11) - Controls = 6 (0-10)   P = 0.5  Duration x past work activity:   - Cases = 6 (0-24) - Controls = 12 (0-25)   P = 0.4  Past leisure-time activity index:   - Cases = 8 (6-9) - Controls = 8 (6-9)   P = 0.8 |
| Pathak and Poston [24]  Prospective cohort | Questionnaires were issued to a consecutive cohort of new patients presenting with an abdominal wall hernia. Data were collected from a single cohort study of patients presenting to a general surgical clinic at an urban general hospital over a 6-month period (January 2003 to June 2003). | Single strenuous or traumatic event. | All cases were clinically diagnosed. Single strenuous or traumatic events were compared to non-strenuous events. | From 133 cases 119 (89%) hernias had been gradual or insidious in onset. There were 14 patients (11%) with a convincing history correlating the symptoms to a single strenuous event. 5 patients of those 14 had no risk factors. One of those 14 stated that they had a strenuous job which involved repetitive heavy lifting or straining. Three patients stated that they believed that their hernias had developed due to strenuous exercise and stretching. Two patients claimed that coughing precipitated their hernia, and two said that their hernia came on suddenly but could not recall what they were doing at the time. The remainder believed that the etiology of their hernias was related to a strenuous event but were unable to identify specifically the causal act. None of these 6 undertook a job that included strenuous physical labour. |
| Ruhl and Everhart [21]  Prospective cohort | NHANES I included  interview, examination, and laboratory data collected from  a national probability sample of the civilian, non-institutionalised  US population.  The NHANES I Epidemiologic Follow-up Study was a longitudinal study of the 14,407 NHANES I participants aged 25–74 years who had been medically examined.  N = 13452 persons remaining for analysis, age 25-74.     - 5316 ♂ - 8136 ♀ | Participants were asked about non-recreational activity (inactive, moderately active, very active). | Non-recreational activity was divided into three categories: low (reference), moderate and high.  HR was adjusted for age. | *Non-recreational physical activity*  Low (N participants, N cases, HR (95% CI)):   - 603 - 36   HR = 1.0  Moderate:   - 2366 - 231   HR = 1.3 (0.92-1.9)  High:   - 2342 - 233   HR = 1.3 (0.90-1.8)  P for trend: 0.42 |
| Smith et al. [25]  Prospective cohort | Consecutive patients presenting with inguinal hernia over a 6-month period were entered into the study.  N = 129 patients, age 20-89.     - 122♂ - 7♀ | Single strenuous event. | All cases were clinically diagnosed. Single strenuous or traumatic events were compared to non-strenuous events. | From 129 cases 120 (93%) hernias had been gradual or insidious in onset. In nine patients (7%) there was a convincing history suggesting an association between a particular muscle strain, groin pain, and the discovery of a groin swelling.  None of these had previously had a hernia repair or appendectomy. Four of the nine patients had a strenuous job, of whom three claimed that the incident had occurred at work. None had any associated medical problems such as chronic chest disease or bladder outflow obstruction. In one patient the diagnosis was made by a doctor within a few days, the remainder being diagnosed between 1 week and 4 years later, median 3.5 weeks. At operation, five of these patients had a direct hernia, three had indirect sacs, and one had not had surgery. One patient described a fall which seemed to precipitate the hernia, while the remainder described lifting strains either at home or work. |
| Snyder and Kearney [27]  Case report | N = 1, a 41-year-old male flight surgeon. | Performing an anti-G straining manoeuvre during high Gz manoeuvres. | N/A | Acute inguinal herniation may occur during high Gz flight with aggressive anti-G straining manoeuvre. |
| **Lifting** | | | | |
| Sanjay and Woodward [28]  Retrospective cohort | Patients who underwent elective inguinal hernia repair under the care of one surgeon, between 1995 and 2004.  Gradual onset: N = 164, aged 20-87   - 158 ♂ - 6 ♀   Sudden onset:  N = 137, aged 19-86   - 131 ♂ - 6 ♀ | Occupation and employment: retired, sedentary, clerical, manual and heavy labour. | In the sudden onset group, predisposing factors predisposing to inguinal herniation were examined. | Type of event predisposing herniation (N = 137):   - Lifting: N = 93 (67.9%) - Coughing: N = 20 (14.6%) - Exercise: N = 14 (10.2%) - Gardening: N = 10 (7.3%) |
| Vad et al. [3]  Prospective cohort | Data from administrative and medical registers in Denmark, that is, the Danish Civil Registration System (CRS), the Employment Classiﬁcation Module (ECM), the Danish National Patient Register (NPR), and the Danish Hernia Database (DHDB).  N= 1 545 987 men, age 18-65     - 22926 lateral hernias - 15877 medial hernias - 1592 pantaloon or unspecified hernias | One ton-year was deﬁned as lifting one ton per day for 1 year. | Zero ton-years was used as a reference compared to 0-1 to 4-17.5 ton-years.  OR was adjusted for age at 1 January each year, socioeconomic status (1-5, categorised with group 1 as reference), region of residence (eight regions based on zip codes), calendar year, and number of follow-up intervals (whole years). | *Lateral inguinal hernia*  Ton-years (N cases):   - 0: 5083   OR (95% CI): 1.00   - 0-1: 3157   OR (95% CI): 1.05 (1.00-1.10)   - 1-2: 3745   OR (95% CI): 1.23 (1.17-1.28)   - 2-3: 3008   OR (95% CI): 1.27 (1.21-1.34)   - 3-4: 2102   OR (95% CI): 1.35 (1.28-1.43)   - 4-17.5: 5719   OR (95% CI): 1.32 (1.27-1.38)  *Medial inguinal hernia*  Ton-years (N cases):   - 0: 4415   OR (95% CI): 1.00   - 0-1: 2286   OR (95% CI): 0.95 (0.90-1.00)   - 1-2: 2623   OR (95% CI): 1.06 (1.00-1.11)   - 2-3: 1895   OR (95% CI): 1.00 (0.94-1.05)   - 3-4: 1193   OR (95% CI): 0.98 (0.91-1.05)   - 4-17.5: 3410   OR (95% CI): 0.99 (0.94-1.05) |
| Vad et al. [3]  Prospective cohort | Data from administrative and medical registers in Denmark, that is, the Danish Civil Registration System (CRS), the Employment Classiﬁcation Module (ECM), the Danish National Patient Register (NPR), and the Danish Hernia Database (DHDB).  N= 1 545 987 men,  Age 18-65     - 22926 lateral hernias - 15877 medial hernias - 1592 pantaloon or unspecified hernias | One frequent-heavy-lifting-year as lifting objects weighing 20 kg or more at least 10 times a day for 1 year. | Zero frequent-heavy-lifting-years was used as a reference compared to frequent-heavy-lifting-years of 0-10 to 40-48.  OR was adjusted for age at 1 January each year, socioeconomic status (1-5, categorised with group 1 as reference), region of residence (eight regions based on post codes), calendar year, and number of follow-up intervals (whole years). | *Lateral inguinal hernia*  Frequent-heavy-lifting-years (n cases):   - 0: 5178   OR (95% CI): 1.00   - 0-10: 3244, 1.06 (1.01-1.11)   OR (95% CI): 1.06 (1.01-1.11)   - 10-20: 3312   OR (95% CI): 1.20 (1.14-1.25)   - 20-30: 2506   OR (95% CI): 1.26 (1.20-1.33)   - 30-40: 2807   OR (95% CI): 1.34 (1.28-1.41)   - 40-48: 5767   OR (95% CI): 1.33 (1.28-1.39)  *Medial inguinal hernia*  Frequent-heavy-lifting-years (n, OR):   - 0, 4515   OR (95% CI): 1.00   - 0-10, 2285   OR (95% CI): 0.94 (0.90-1.00)   - 10-20, 2294   OR (95% CI): 1.03 (0.97-1.08)   - 20-30, 1568   OR (95% CI): 0.95 (0.89-1.01)   - 30-40, 1706   OR (95% CI): 1.02 (0.96-1.08)   - 40+, 3460   OR (95% CI): 1.00 (0.95-1.06) |
| Vad et al. [6]  Prospective cohort | Data from the Musculoskeletal  Research Database (MRD) at the Danish Ramazzini Centre was used.  The MRD originally contained questionnaire data from 39590 persons, who had participated in at least one of nine studies of Danish working populations, which were  conducted from 1993 to 2004. Since then, questionnaire data  from 4325 participants in a general practice study from 2008  have been added, including 1858 men who reported that they  were occupationally active.  N = 17967 men aged 18-65 (birth years 1932-1990, both years included).     - 382 lateral hernia repairs - 314 medial hernia repairs | The total load  lifted per day was categorised as 0 (reference), > 0 -<1000  and 1000 -≤ 4900kg/day. | The total load lifted per day of 0 was used as a reference compared to >0-<1000 and 1000-≤4900 kg/day.  HR_adj_ was adjusted for age at the start of follow-up  HR_fully adj_ was adjusted for age at the start of follow-up, body mass index, leisure-time physical activity, smoking status and year of data collection in the original study. | *Lateral inguinal hernia*  Total load lifted per day (kg/day): 0 (N person years, N cases) (reference):   - 68156 - 117   HR_adj_: 1.00  HR_fully adj_: 1.00  Total load lifted per day (kg/day): 0-1000:   - 74266 - 141   HR_adj_ (95% CI): 1.14 (0.89-1.46)  HR_fully adj_ (95% CI): 1.14 (0.88-1.61)  Total load lifted per day (kg/day): 1000-≤4900:   - 58888 - 124   HR_adj_ (95% CI): 1.32 (1.02-1.72)  HR_fully adj_ (95% CI): 1.22 (0.89-1.66)  *Medial inguinal hernia*  Total load lifted per day (kg/day): 0 (N person years, N cases) (reference):   - 68156 - 109   HR_adj_: 1.00  HR_fully adj_: 1.00  Total load lifted per day (kg/day): 0-1000:   - 74266 - 114   HR_adj_ (95% CI): 0.99 (0.75-1.28)  HR_fully adj_ (95% CI): 1.00 (0.76-1.32)  Total load lifted per day (kg/day): 1000-≤4900:   - 58888 - 91   HR_adj_ (95% CI): 1.03 (0.77-1.37)  HR_fully adj_ (95% CI): 0.94 (0.66-1.32) |
| Vad et al. [6]  Prospective cohort | Data from the Musculoskeletal  Research Database (MRD) at the Danish Ramazzini Centre was used.  The MRD originally contained questionnaire data from 39590 persons, who had participated in at least one of nine studies of Danish working populations, which were  conducted from 1993 to 2004. Since then, questionnaire data  from 4325 participants in a general practice study from 2008  have been added, including 1858 men who reported that they  were occupationally active.  N = 17967 men aged 18-65 (birth years 1932-1990, both years included).     - 382 lateral hernia repairs - 314 medial hernia repairs | The daily frequency  of lifting loads weighing ≥20kg was categorised as <2  (reference), 2-<11 and 11-≤89lifts/day. | The daily frequency of lifting loads weighing ≥20kg <2 lifts/day was used as a reference compared to 2-<11 and 11-<89 lifts/day.  HR_adj_ was adjusted for age at the start of follow-up  HR_fully adj_ was adjusted for age at the start of follow-up, body mass index, leisure-time physical activity, smoking status and year of data collection in the original study. | *Lateral inguinal hernia*  Frequency of lifting loads weighing 20+ kg (lifts/day): 0-2 (N person years, N cases) (reference):   - 73902 - 125   HR_adj_: 1.00  HR_fully adj_: 1.00  Frequency of lifting loads weighing 20+ kg (lifts/day): 2-11   - 63327 - 127   HR_adj_: 1.20 (0.94-1.54)  HR_fully adj_: 1.19 (0.91-1.54)  Frequency of lifting loads weighing 20+ kg (lifts/day): 11-89   - 64082 - 130   HR_adj_: 1.31 (1.01-1.67)  HR_fully adj_: 1.21 (0.91-1.62)  *Medial inguinal hernia*  Frequency of lifting loads weighing 20+ kg (lifts/day): 0-2 (N person years, N cases) (reference):   - 73902 - 114   HR_adj_: 1.00  HR_fully adj_: 1.00  Frequency of lifting loads weighing 20+ kg (lifts/day): 2-11   - 63327 - 105   HR_adj_ (95% CI): 1.09 (0.84-1.43)  HR_fully adj_ (95% CI): 1.04 (0.82-1.32)  Frequency of lifting loads weighing 20+ kg (lifts/day): 11-89   - 64082 - 95   HR_adj_ (95% CI): 1.03 (0.78-1.36)  HR_fully adj_ (95% CI): 0.86 (0.64-1.14) |
| Standing/walking | | | | |
| Vad et al. [3]  Prospective cohort | Data from administrative and medical registers in Denmark, that is, the Danish Civil Registration System (CRS), the Employment Classiﬁcation Module (ECM), the Danish National Patient Register (NPR), and the Danish Hernia Database (DHDB).  N= 1 545 987 men, aged 18-65     - 22926 lateral hernias - 15877 medial hernias - 1592 pantaloon or unspecified hernias | One standing-year was deﬁned as standing/walking 6 hours per day for 1 year—for example, standing/walking 3 hours per day for 1 year was given a value of 0.5 standing-years. | Zero standing-years was used as a reference compared to standing-years from 0-2 till 5-6.  OR was adjusted for age at 1 January each year, socioeconomic status (1-5, categorised with group 1 as reference), region of residence (eight regions based on post codes), calendar year, and number of follow-up intervals (whole years). | *Lateral inguinal hernia*  Standing-year (N cases):   - 0: 5060   OR (95% CI): 1.00   - 0-2: 4425   OR (95% CI): 1.07, (1.02-1.11)   - 2-3: 2681   OR (95% CI): 1.17, (1.11-1.23)   - 3-4: 4472   OR (95% CI): 1.31, (1.25-1.37)   - 4-5: 4564   OR (95% CI): 1.36, (1.30-1.42)   - 5-6.1: 1675   OR (95% CI): 1.43, (1.34-1.51)  *Medial inguinal hernia*  Standing-year (N, OR):   - 0: 4403   OR (95% CI): 1.00   - 0-2: 3005   OR (95% CI): 0.93, (0.88-0.97)   - 2-3: 1781   OR (95% CI): 0.96, (0.90-1.02)   - 3-4: 2953   OR (95% CI): 1.06, (1.01-1.12)   - 4-5: 2690   OR (95% CI): 1.00, (0.95-1.06)   - 5-6.1: 1053   OR (95% CI): 1.14, (1.06-1.23) |
| Vad et al. [6]  Prospective cohort | Data from the Musculoskeletal  Research Database (MRD) at the Danish Ramazzini Centre was used.  The MRD originally contained questionnaire data from 39590 persons, who had participated in at least  one of nine studies of Danish working populations which were  conducted from 1993 to 2004. Since then, questionnaire data  from 4325 participants in a general practice study from 2008  have been added, including 1858 men who reported that they  were occupationally active.  N = 17967 men aged 18-65 (birth years 1932-1990, both years included).     - 382 lateral hernia repairs - 314 medial hernia repairs | Standing/walking was categorised as < 4 (reference), 4 –<6 and 6 –≤ 7.3 hours/day. | Standing/walking for <4 hours/day was used as a reference compared to 4-<6 hours a day and 6-<7.3 hours/day  HR_adj_ was adjusted for age at the start of follow-up  HR_fully adj_ was adjusted for age at the start of follow-up, body mass index, leisure-time physical activity, smoking status and year of data collection in the original study. | *Lateral inguinal hernia*  Standing/walking (hours/day): 0-4 (n person years, n cases) (reference):   - 91010 - 149   HR_adj_: 1.00  HR_fully adj_: 1.00  Standing/walking (hours/day): 4-6   - 21279 - 32   HR_adj_ (95% CI): 0.88 (0.60-1.30)  HR_fully adj_ (95% CI): 0.89 (0.61-1.31)  Standing/walking (hours/day): 6-7.3   - 89022 - 201   HR_adj_ (95% CI): 1.42 (1.15-1.76)  HR_fully adj_ (95% CI): 1.45 (1.12-1.88)  *Medial inguinal hernia*  Standing/walking (hours/day): 0-4 (N person years, N cases) (reference):   - 91010 - 130   HR_adj_: 1.00  HR_fully adj_: 1.00  Standing/walking (hours/day): 4-6   - 21279 - 33   HR_adj_ (95% CI): 1.04 (0.71-1.53)  HR_fully adj_ (95% CI): 1.06 (0.72-1.56)    Standing/walking (hours/day): 6-7.3   - 89022 - 151   HR_adj_ (95% CI): 1.20 (0.95-1.53)  HR_fully adj_ (95% CI): 1.25 (0.93-1.66) |
